# The use of external change agents to promote quality improvement and organizational change in healthcare organizations: a systematic review

**DOI:** 10.1186/s12913-018-2856-9

**Published:** 2018-01-25

**Authors:** Esra Alagoz, Ming-Yuan Chih, Mary Hitchcock, Randall Brown, Andrew Quanbeck

**Affiliations:** 1 Wisconsin Surgical Outcomes Research Program (WiSOR), University of Wisconsin-Madison, 600 Highland Ave, Madison, WI 53792-1690 USA; 2 Department of Clinical Sciences, University of Kentucky College of Health Sciences, Room 209 Wethington Building, 900 South Limestone Street, Lexington, KY 40536-0200 USA; 3 Senior Academic Librarian, Ebling Library for the Health Sciences, University of Wisconsin- Madison, Madison, USA; 4 Department of Family Medicine, University of Wisconsin School of Medicine & Public Health, 1100 Delaplaine Ct, Madison, WI 53715 USA; 5 Department of Family Medicine & Community Health, Research Scientist- Center for Health Enhancement Systems Studies, Department of Industrial & Systems Engineering, University of Wisconsin-Madison, 4115 Mechanical Engineering Building, 1513 University Avenue, Madison, WI 53706 USA

**Keywords:** External change agents, Quality improvement, Organizational change, Practice facilitation, Academic detailing

## Abstract

**Background:**

External change agents can play an essential role in healthcare organizational change efforts. This systematic review examines the role that external change agents have played within the context of multifaceted interventions designed to promote organizational change in healthcare—specifically, in primary care settings.

**Methods:**

We searched PubMed, CINAHL, Cochrane, Web of Science, and Academic Search Premier Databases in July 2016 for randomized trials published (in English) between January 1, 2005 and June 30, 2016 in which external agents were part of multifaceted organizational change strategies. The review was conducted according to PRISMA guidelines. A total of 477 abstracts were identified and screened by 2 authors. Full text articles of 113 studies were reviewed. Twenty-one of these studies were selected for inclusion.

**Results:**

Academic detailing (AD) is the most prevalently used organizational change strategy employed as part of multi-component implementation strategies. Out of 21 studies, nearly all studies integrate some form of audit and feedback into their interventions. Eleven studies that included practice facilitation into their intervention reported significant effects in one or more primary outcomes.

**Conclusions:**

Our results demonstrate that practice facilitation with regular, tailored follow up is a powerful component of a successful organizational change strategy. Academic detailing alone or combined with audit and feedback alone is ineffective without intensive follow up. Provision of educational materials and use of audit and feedback are often integral components of multifaceted implementation strategies. However, we didn’t find examples where those relatively limited strategies were effective as standalone interventions. System-level support through technology (such as automated reminders or alerts) is potentially helpful, but must be carefully tailored to clinic needs.

## Background

Change agents play an essential role in healthcare organizational change efforts. In his influential book *Diffusion of Innovations* [1], Everett Rogers introduces the concept of change agents as people who “introduce innovations into a client system that they expect will have consequences that will be desirable, direct, and anticipated.” Change agents can be internal to a client organization (for example, organizational leaders who support change; project champions who actively promote change; or organizational “opinion leaders” who, through their endorsement, promote change implicitly). This paper focuses on the role of *external change agents*. The Consolidated Framework for Implementation Research, a compendium of terms and constructs used in implementation research, defines external change agents as: “Individuals who are affiliated with an outside entity who formally influence or facilitate intervention decisions in a desirable direction. They usually have professional training in a technical field related to organizational change science or in the technology being introduced into the organization. This role includes outside researchers who may be implementing a multisite intervention study and other formally appointed individuals from an external entity (related or unrelated to the organization); e.g., a facilitator from a corporate or regional office or a hired consultant.” [2] In the research literature on organizational change in healthcare, external change agents go by various names, including (but not limited to) *facilitator, coach, preceptor, consultant,* and *mentor*. A compilation of discrete implementation strategies lists tactics such as “use an improvement/implementation advisor,” “Conduct educational outreach visits,” “Conduct ongoing training,” and “External facilitation,” all of which are examples of implementation strategies where external change agents play an essential role [3].

External change agents are fundamental to many implementation strategies. Often, the external change agent is the individual responsible for delivering the implementation strategy, and is thus largely responsible for fidelity to the intended strategy and its ultimate success in achieving desired organizational changes. This systematic review is designed to examine the role that external change agents have played in promoting organizational change in healthcare. We have chosen to focus our analysis on organizational change efforts within primary care clinics which are often relatively small, community-based clinics that generally lack the kind of internal quality improvement resources that are relatively common in larger healthcare organizations (including designated, internal change agents). However, we did not exclude any study solely based on the size of practice. Ultimately, this systematic review seeks to provide useful information to health services and implementation researchers in designing effective implementation strategies that involve external change agents to promote change in primary care settings and community- based clinics.

## Methods

### Review design

The protocol for this systematic review was designed in accordance with PRISMA guidelines. We engaged a research librarian (M.H.) to assist in developing the search strategy. The librarian ran a number of search strategies to fine-tune the search terms to eliminate off-topic results. This stage consisted of an iterative review and revision process between the first author and the librarian to refine the list of abstracts. The list of search results included 477 abstracts.

### Data sources and search strategy

With the assistance of the librarian, we searched PubMed, CINAHL, Cochrane, Web of Science, and Academic Search Premier Databases in July 2016 for studies published between January 1, 2005 and June 30, 2016. We incorporated MeSH terms, and CINAHL headings to refine our search results. The index terms and text words we used in our search consisted combinations of the following: *quality improvement* OR *change* OR *process improvement* OR (*organizational improvement* OR *organizational change*) AND (*outpatient clinics* or *primary care* OR *drug use* OR *drug abuse* OR *opioid* OR *substance abuse* OR *addiction*) AND (*coaching* or *facilitation* or *mentoring* OR precept* or *consulting* OR academic detail*). Originally we thought we would include addiction treatment clinics, but later excluded them because they operate very differently from primary care clinics.

We applied filters for study type, omitting articles classified as case reports, clinical conferences, comments, editorials, letters, lectures, meta-analyses, opinion pieces, or reviews.

We also consulted with field experts to ask about studies that they would suggest we include in our review. The suggestions and reference lists forwarded by the experts were reviewed by the first author according to the search criteria and included in the final list of 339 abstracts.

### Inclusion and exclusion criteria

We included randomized controlled trials that investigate process improvement activities in healthcare organizations (general practices, primary care practices, private practices, and outpatient clinics) through external change agents. Because external change agents are referred by a number of different titles, we included studies that provide facilitating, coaching, mentoring, precepting, and academic detailing to the organizations’ staff members (physicians, nurses, administrators, etc.) through an external change agent. We focused our review on peer- reviewed clinical trials published since 2005 that measure the effects of 1 or more interventions.

We excluded studies describing patient coaching, as we wanted to focus on organizational change rather than individual health behavior change. We also excluded non-randomized studies, studies without a control group, and studies without the full text available in English, as well as reviews, conference proceedings, qualitative studies, and opinion pieces. In general, the initiatives targeted larger, clinic-wide changes rather than changes at the level of the individual practitioner.

### Study selection and data extraction

We selected articles in 2 phases. In phase 1, two of the authors (EA and MC) independently screened titles and abstracts of the search results after removing duplicates based on the inclusion and exclusion criteria. We obtained full articles for the abstracts that we identified as relevant. After a second round of elimination that included reviewing full text articles based on the inclusion criteria, the final set of studies were identified. A number of clinical trials were excluded because they did not report on the effectiveness of the interventions. For phase 2, we independently reviewed the final list of full-text articles for the following variables: 1. intervention type, 2. setting, 3. study design, 4. intervention components, 5. background of change agents, 6. background of settings, 7. intervention duration and frequency of contact, 8. outcome measures, and 9. significance. A final review of the search results was conducted by the senior author (AQ) to finalize the list of included studies and the set of study variables and outcomes used to summarize them. Table 1 summarizes these variables.

**Table 1:** Summary of studies included in the review

Source	Country	Study Design	Background of external change agent	Study Arms	Intervention strategies	Primary Outcomes	Results
Aspy et al., 2008 [25]	USA	Cluster RCT of 16 small-sized practices	Quality improvement agent	Intervention arm (8 practices): feedback with benchmarking, academic detailing, assistance of practice enhancement Vs. usual care (8 practices)	1. Audit and feedback 2. Academic detailing 3. Practice facilitation	- proportion of mammograms recommended -rate of performed mammograms	-Intervention arm offered significantly more mammograms than control arm (*p* = .043) − 52% in intervention arm completed mammograms vs. 35% in control arm (*P* < .015)
Bertoni et al. 2009 [26]	USA	Cluster RCT: 29 practices in intervention and 32 in control (mixed sizes of small and middle)	physician-investigator	Both arms: treatment guidelines, intro lecture, 1 feedback report, 4 academic detailing visits Intervention arm only: Personal digital assistant-based decision support	1. Academic detailing 2. System support (Personal digital assistant-based decision support)	- screening rate for lipid levels - appropriate management of lipid levels - appropriate drug prescription - overtreatment	The screening rate for lipid levels increased in intervention but was not significant (*p* = .22). Appropriate management of lipid levels decreased in both arms but the difference favored intervention arm (*p* = .01). Appropriate drug prescription decreased in both arms (*p* = .37). Overtreatment declined from 6.6% to 3.9% in intervention but rose in control from 4.2% to 6.4% (*p* = .01).
Clyne et al., 2015 [30]	Ireland	Cluster RCT: 21 mid-sized GP practices	pharmacist	Intervention (11 practices): academic detailing; web-based review of medicines, tailored patient info leaflets Control (10 practices): Usual care + simple pt.-level feedback	1. Academic detailing 2. System support (Web-based treatment options) 3. Provision of educational materials (Patient information leaflets)	-proportion of patients with inappropriate prescribing -mean no. of potentially inappropriate prescriptions	-Patients in intervention group had lower odds of inappropriate prescribing (*p* = .02) -Mean no. of inappropriate prescriptions also significantly lower in intervention group (*p* = .02)
Dickinson et al., 2014 [18]	USA	Cluster RCT of 40 small to mid-sized PCPs	Quality improvement agent	3 arms: 1. Practice facilitation for 6 mo. using reflective adaptive process (RAP) 2. Practice facilitation for up to 18 months using continuous quality improvement (CQI) 3. Self-direction (SD) practices with model info and resources—no facilitation	1. Practice facilitation 2. Audit and feedback	- diabetes quality measures (chart audits) - Practice Culture Assessment surveys of clinicians & staff	- Quality of diabetes care improved in all 3 groups (all *P* < .05). Improvement was greater in CQI practices compared with both SD practices (*P* < .0001) and RAP practices (*P* < .0001), and in SD practices vs. RAP practices (*P* < .05). - Change Culture scores in RAP practices showed trend of improvement at 9 mo. (*P* = .07) but decreased below baseline at 18 months (*P* < .05), and Work Culture scores decreased from 9 to 18 months (*P* < .05). In CQI and SD practices, culture scores were stable over time.
Dignan et al., 2014 [28]	USA	Cross-over cluster RCT of 66 mixed-size PC practices, 33 per arm	Local people who knew primary care were trained in academic detailing	Intervention (33 practices): “Early” clinics received academic detailing for 6 months Control (33 practices): “Delayed” clinics received no intervention until after data were collected at 6 mo. Then delayed clinics received the academic detailing intervention the “early” clinics had received	1. Academic detailing	- recommendations for screening - completed screenings	- No increase in recommendations for screening - Rates of completed screenings were higher for all practices for the most common screening methods (colonoscopy and fecal occult blood testing), though rates of completed colonoscopy were higher in early clinics vs. delayed (*p* = 0.01).
Engels et al., 2006 [19]	Netherlands	Cross-over cluster RCT of 49 large PC practices	Trained outreach visitors	Intervention arm (26 practices): Assessment of practice mgmt. Using VIP; detailed written and oral feedback; workbook with CQI tools; trained facilitator used in 5 monthly team meetings; use of QI cycles; transfer of task from facilitator to team. Control (23 practices): Assessment using VIP with written feedback delivered in 1-h. meeting	1. Audit and feedback 2. Practice facilitation	-the number of improvement projects undertaken -the number of improvement steps taken for each QI project (to measure quality) -the number of self-defined objectives met	- Intervention group practices had significantly better results on all 3 outcomes vs. control
Feldstein et al., 2006 [29]	USA	Cluster RCT of 15 large sized clinics from one HMO	physicians	Intervention 1 (7 clinics): alerts in EHR + group academic detailing Intervention 2 (8 clinics): Alerts only	1. Academic detailing 2. System support (EMR alerts)	- the rate of interacting prescriptions	Reduction in the interacting medication prescription rate resulting in a 14.9% relative reduction at 12 months (*p* < .001). Group academic detailing did not enhance alert effectiveness.
Hennesy et al., 2006 [31]	USA	Cluster RCT with 93 PC providers (clinic size NA)	clinical pharmacist	Intervention (39 providers): academic detailing visit, provider-specific data, provision of educational materials, Control: (54 providers): no intervention	1. Academic detailing 2. Audit and feedback 3. Provision of educational materials	- the rate of blood pressure measurement below 140/90 mmHg	No significant difference was detected between study arms
Hogg et al., 2008 [36]	Canada	Match-paired Cluster RCT of 54 small to mid-sized PC practices	Masters level nurses trained in facilitation	Intervention arm (27 practices): monthly visits + delivery of preventive interventions (goal setting, learning about tools, planning for reaching goals, adapting) Control (27 practices): no services from facilitator	1. Audit and feedback 2. Practice facilitation	- Practices’ delivery of preventive maneuvers, measured by preventive performance indices from chart reviews and patient survey data.	No difference was detected between the trial’s arms for the primary outcome.
Lowrie et al., 2014 [22]	UK	Cluster RCT of 31 small PC practices	pharmacist	Intervention (16 practices): org support (id patients, id barriers to prescribing change, plan for overcoming barriers, plans for indiv patients) + 3 face-to-face mtgs Control (15 practices): usual care	1. Audit and feedback 2. Academic detailing	-the proportion of patients achieving cholesterol targets	Intervention patients were significantly more likely to have cholesterol at target (69.5% vs 63.5%; OR 1.11, CI 1.00–1.23; *p* = 0.043) as a result of improved simvastatin prescribing.
Magrini et al., 2014 [23]	Italy	Two separate Cluster RCTs: TEA vs SIDRO—115 PC groups in both studies (clinic size NA)	pharmacist	Intervention: Facilitator has biannual 3–4 h. meetings with audit & feedback and problem-based learning In both TEA and SIDRO trials, 57 primary care groups in arm 1 (TEA: benign prostatic hyperplasia; SIDRO: prulfloxacin) and 58 in arm 2 (TEA: osteoporosis; SIDRO: basmidapine)	1. Academic detailing (biannual 3–4 h meetings) 2. Audit and feedback	-changes in the six-months prescription of targeted drugs: -TEA: Prescription of alfuzosin compared to tamsulosin and terazosin. (disease oriented) -SIDRO: Single drug oriented, pharmacist as the main actor or facilitator of an easy-to-use evidence synthesis, and has more clear-cut outcomes based on prescribing of a single drug	In the TEA trial, one of the four primary outcomes showed a reduction (prescription of alfuzosin compared to tamsulosin and terazosin in benign prostatic hyperplasia: prescribing ratio 28.5%, *p* = 0.03). Another primary outcome (prescription of risedronate) showed a reduction at 24 and 48 months (27.6%, *p* = 0.02; and 29,8%, *p* = 0.03), but not at six months (25.1%, *p* = 0.36). In the SIDRO trial both primary outcomes showed a statistically significant reduction (prescription of barnidipine 29.8%, *p* = 0.02; prescription of prulifloxacin 211.1%, *p* = 0.04), which persisted or increased over time. Conclusion: Intervention worked better for more straightforward, single-drug trial. *Note: Randomization was to arm that covers one disease or another (for TEA) or one drug or another (for SIDRO). Everybody got same intervention.*
Mold et al., 2008 [17]	USA	Individual RCT (small to mid-sized practices)	Practice facilitator and IT person	Intervention (12 clinics): One clinician/nurse team per practice received performance feedback peer-to-peer education (academic detailing), a practice facilitator, and computer (IT) support) Control (12 clinics): Performance feedback and benchmarking alone	1. Audit and feedback 2. Academic detailing 3. Practice facilitation 4. System support (automated reminders)	- Standing orders: protocols or policies that authorize staff to deliver services (measure is 50%use) - Reminders: paper based or electronic (measure is 50%use) -Wellness visits devoted to providing preventive services. (measure is 50%use)	- Standing orders: 9/14 vs 1/8 (*p* = .02) - Reminders: 6/8 vs 1/4 (*p* = .10) - Visits: 5/16 vs 2/10 (*p* = .53) (P is calculated by binomial proportions test) Authors’ conclusion: The multicomponent strategy increased implementation of evidence-based processes to a greater extent than performance feedback and benchmarking alone.
Mold et al., 2014 [32]	USA	Cluster RCT in 43 (mixed sizes) PC practices from 3 research networks	Practice facilitator	All practices rec’d performance feedback, academic detailing, summaries of guidelines, and a toolkit of asthma tests and action plan templates. 4 arms: 1. Practice facilitation (PF) alone (10 practices): visits ½ day/week or 1 day/every other week for 6 months. 2. Local learning collaboratives (LLCs) (10 practices): monthly meetings among practices to review data and plans 3. PF + LLC (12 practices) 4. Control (11 practices)	1. Audit and feedback 2. Academic detailing 3.Practice facilitation 4. Local Learning collaboratives (LLC)	Adherence to 6 recommendations: - Documentation of severity assessment - Assessment of exposure to environmental triggers, -Assessment of level of control, -Prescription of controller medications -Written asthma action plan, -Planned asthma visits	- Statistically significant adoption rates at each arm: Control group: 2 out of 6 recommendations Practice Facilitation: 3 out of 6 LLC: 4 out of 6 PF + LLC: 5 out of 6 PF practices improved assessment of asthma severity and assessment of asthma level of control (*p* = .005) - LLCs are not significantly effective.
Naughton et al., 2009 [21]	Ireland	Cluster RCT (98 GP clinics) (clinic size NA)	Pharmacist	1. Audit and feedback via postal bulletin containing educational materials (50 GP clinics) 2. Audit and feedback via postal bulletin containing educational materials + Academic detailing (48 GP clinics)	1. Academic detailing 2. Provision of educational materials (postal bulletin)	Prescription data pulled from national prescribing database	Antibiotic prescribing was significantly reduced in both groups, suggesting that receiving prescribing feedback was effective in reducing prescribing rates; however, there was no significant difference reported between the AF and AD groups.
Ornstein et al., 2010 [33]	USA	2-arm cluster RCT (32 small-sized PC clinics)	A physician and a nurse (PIs)	1. Quality improvement (QI) intervention combining EMR based audit and feedback, practice site visits for academic detailing and participatory planning (4 half day site visits over 2 years), and “best-practice” dissemination on CRC screening delivered via bi-annual in person meetings of participants vs. 2. Control (usual care)	1. Audit and feedback 2. Academic detailing 3. Practice facilitation	Proportion of active patients aged 50–75 up to date with CRC screening; proportion of active patients among those not up to date with CRC screening having screening recommended within past year.	Patients 50–75 years in intervention practices exhibited significantly greater improvement in being up-to-date with CRC screening than patients in control practices (*p* < .001; adjusted difference 4.9%); recommendations for screening also improved in the intervention group (*p* < .001; adjusted difference 7.9%).
Ornstein et al., 2013 [34]	USA	Delayed intervention, group-randomized trial of 19 small to mid-sized PC clinics.	Physician (PI)	Intervention consisted of quarterly feedback reports; 4 in-person site visits for academic detailing and participatory planning; and 2 in-person meetings of participants for networking and sharing of best practices.	1. Academic detailing 2. Audit and feedback 3. Practice facilitation	Improving screening rates for problem alcohol use, provision of brief interventions, and use of pharmacotherapy for patients with diabetes and/or hypertension	Patients in early-intervention practices were significantly more likely than patients in delayed-intervention practices to have been screened ((odds ratio [OR] = 3.30, 95% CI [1.15, 9.50]) and more likely to have been provided a brief intervention (OR = 6.58, 95% CI [1.69, 25.7].) The intervention had little effect on use of pharmacotherapy for alcohol use disorders.
Parchman et al., 2013 [20]	USA	Stepped-wedge study design with block randomization of practices in groups of 10 (40 small PC clinics total).	Quality Improvement experts	Practice facilitation with integral audit and feedback. Facilitator held a minimum of six one-hour team meetings within each practice over a 12-month period	1. Audit and feedback 2. Practice facilitation (CCM model) 3. System support	Assessment of Chronic Illness Care (ACIC) survey score, a survey instrument designed to measure concordance with tenets of the chronic care model	- Practices randomized to early intervention showed a significant improvement in ACIC scores (*p* < 0.05) compared to the delayed intervention (control) practices. This increase was sustained after one year.
Rognstad et al., 2013 [24]	Norway	2-arm cluster RCT (449 GP providers) (mixed sizes of clinics)	GP physicians associated with a university (including investigators)	Intervention consisted of 2 academic detailing visits and review of a personalized audit and feedback report of providers’ potentially inappropriate prescriptions for older adults, plus an in-person full day workshop. vs. 2. GPs in the control group were assigned to another educational intervention targeting antibiotic prescribing practice for respiratory tract infections.	1. Academic detailing 2. Audit and feedback (mailed report)	Percentage of patients with potentially inappropriate prescriptions (PIPs) – based on thirteen explicit criteria.	A reduction relative to baseline of 10.3% in PIPs per 100 patients aged ≥70 years was obtained in the intervention group compared to the control group.
Sheffer et al., 2012 [27]	USA	Two-arm cluster RCT (49 PC small sized clinics)	Study physician and outreach specialist (health educator)	Control condition: clinic is provided with a manual that describes the roles and responsibilities required of members of the healthcare delivery team to successfully implement a clinic-based fax referral program. In addition, clinics receive audit/feedback reports and access to educational materials. Vs. Intervention condition: in addition to above, clinics received On-site training at launch and 6 months (by outreach specialist to key clinic staff); Telephone check-in and performance feedback at 1 and 9 months (by outreach specialist to clinic manager); Telephone check-in and performance feedback at 3, 6, and 9 months (by study physician to clinic physician leader). .	1. Academic detailing 2. Audit and feedback 3. Practice facilitation 4. Provision of educational materials	-Number of referrals -Number of quality referrals	- Mean number of post-intervention referrals/clinician to the Wisconsin Tobacco Quitline was 5.6 times greater in the intervention group (*p* = 0.001). -Number of quality referrals was higher in intervention group (*p* = 0.001)
Smidth et al., 2013 [35]	Denmark	2-group Cluster RCT with additional non-randomized control group (clinic size NA)	Trained QI facilitators	Intervention group: practices were invited to participate in four two-and-a-half-hour sessions. The Breakthrough Series was used as a framework for implementation. One facilitator visited each practice to address challenges encountered in pursuing their goals. Control: standard governmental implementation protocols.	1. Audit and feedback 2. Practice facilitation (CCM model) (4 sessions) 3. System support	- Adherence to disease management programs for chronic obstructive pulmonary disease, measured using the Patient-Assessment-of-Chronic-Illness-Care (PACIC) instrument.	There was a statistically significant change in the PACIC score in the intervention group than in the control group (intervention effect = 0.12 [95% CI: 0.00;0.25].
Varonen et al., 2007 [13]	Finland	2 group Cluster RCT (30 large sized PC clinics)	General practice physicians	Intervention group: Academic detailing Our modification of AD included use of information sources, feedback of own practices and visits of external experts. Control: Problem-based learning, a clinician education method based on group work facilitated by a local GP tutor that utilized case scenarios, information retrieval and reflection.	1. Academic detailing 2. Problem-based learning	The effect of guideline implementation on acute maxillary sinusitis management	Implementation of guidelines produced only modest changes in the management of AMS. There were no significant differences between academic detailing and problem based learning education methods..

*PC* Primary Care, *GP* General Practice, *RCT* Randomized Controlled Trial

### Data analysis

Because the research domains, study methods, and measures were disparate, we could not perform the necessary data pooling for a meta-analysis. We performed a qualitative synthesis to categorize how study interventions –specifically the methods of change implementation-- affected the outcomes of the research. We analyzed the multifaceted interventions employed in each study under 5 separate categories based on our qualitative synthesis.

Definitions of Types of Interventions

1. *Academic detailing*. Academic detailing is delivered by a trained professional in a face-to-face meeting at varying intervals. These are informative sessions aimed at improving a provider’s knowledge on a specific subject matter and usually aim to influence prescribing patterns. Detailers don’t instruct clinicians to practice in a certain way or provide hands-on facilitation of organizational change.
2. *Audit and feedback*. Audit and feedback is a summary of clinic and/or provider performance delivered to healthcare professionals to improve their practices. Although audit and feedback is often delivered in face-to-face sessions, some studies also send the reports via regular or electronic mail. Benchmarking, which is comparing a clinician’s practice with a standard, is included under this component.
3. *Provision of educational materials*. This component involves types of educational materials that are delivered by mail or electronically, or in-person. Provision of educational materials is often included as a component in interventions that feature external change agents.
4. *Practice facilitation (or coaching)*. Practice facilitation involves an external change agent who visits sites on a regular basis to assist with implementing changes and answering change-related questions. The change agent, unlike an academic detailer, follows up with the clinic on a regular basis to provide individualized feedback.
5. *System support*. If the intervention includes an aspect of technical assistance, we identified that as system support (EHR systems, billing systems, IT support, etc.).

*Authors’ note: the terms “academic detailing,” “facilitation,” and “coaching” are not well defined nor rigorously applied in the research literature. In this review, ‘practice facilitation’ includes facilitating, coaching, and mentoring. We have supplied general terms for reference here In the study arms column in* Table 1*, we specify the frequency of contact the external change agent had with clinics.*

### Quality assessment

We also assessed the quality of clinical trials included in this study. The lead author (EA) assessed risk for bias using the Cochrane Handbook for Systematic Reviews of Interventions for randomized clinical trials. The senior author (AQ) randomly selected 4 of the articles to rate for risk of bias. Studies were rated on five elements affecting risk of bias and each study received an overall quality rating of high, low, or unclear risk of bias. Of the 4 randomly selected articles, the co-authors agreed on 85% (17/20) of their ratings in the five subdomains, indicating high concordance in ratings of risk of bias. Of note, the search terms we employed led us to review only articles with rigorous study designs, meaning those with randomization to treatment and control groups. Hence, no studies were excluded on the basis of risk of bias.

## Results

### Search outcome

**Fig. 1:**
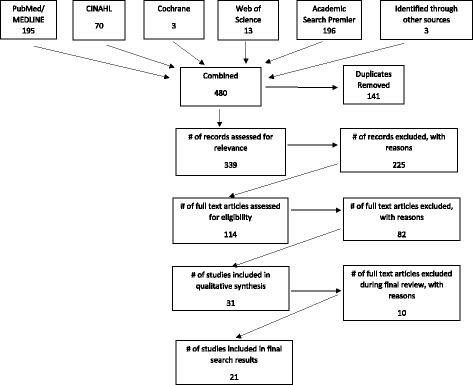
Flowchart

The initial search of 5 databases identified 477 articles published from January 1, 2005 to June 31, 2016 (Fig. 1). Three additional titles were identified through other sources (i.e. expert feedback). One hundred forty-one of these articles were duplicates. We screened the remaining 339 titles and abstracts for relevance. Of these, 226 articles were excluded because they did not meet the inclusion criteria. Full text articles of 113 studies were reviewed. Thirty-four of these studies were selected for inclusion. After the final selection, we examined the studies in more detail. During this process, we identified a number of other articles that were not eligible. The reasons for exclusion are as follows. The first article we excluded did not report on the effectiveness of the interventions but reported on the parts of a larger clinical trial. We searched for the larger trial that the study was part of. However, it was published before 2005 [4]. The second study investigated the acceptability of the intervention and did not report on effectiveness [5]. The third study was eliminated because it reported the cost analysis of a randomized controlled study (RCT) conducted in 2005 [6]. We excluded a group of studies that were conducted in disparate healthcare and community settings outside of primary care, including intensive care units, dental care, and addiction treatment clinics [7–13]. After reading through the articles, we decided to exclude two studies conducted in developing nations [14, 15] to make sure the results we report are compatible in terms of context. We also excluded one study that upon further examination proved not to be a RCT [16]. We extracted data from a final set of 21 studies.

### Study, clinic, and intervention characteristics

Table 1 summarizes and details the study characteristics of the set of 21 studies that are included in this review. The studies represented 9 countries, with most studies occurring in the United States. Most of the included studies were cluster RCTs in which clinics were randomized. All included studies focused on Primary Care Practices (PCP) and General Practices (GP). Hence, the target clients were mostly physicians and nurses.

The studies reported on a number of interventions, combining the five components described previously. Table 1 lists details of the interventions for each study. Most of the studies focused on changing policies regarding chronic conditions care. Seventeen studies reported patient outcomes while 4 studies reported on measures developed by the investigators (such as surveys, self-defined objectives, etc.) [17–20]. The studies reporting on patient outcomes pulled the data from an external electronic database (such as an electronic health record). The majority of studies measured the effects of intervention components as a single intervention.

### Quality of included studies and risk for bias

The methodologic quality of included randomized clinical trials is available in Table 2. Most of the studies had low risk of selection and performance bias. The participants and staff were unblinded to the interventions in all cases. However, we decided that lack of blinding would have minor impact on study outcomes because most studies analyzed the intervention effects on patient outcomes while randomizing at the clinic level. A number of unblinded studies utilized self-assessments (either clinician or patient) as outcome measurements. Those cases are reported as high risk. Overall, majority of the clinical trials in this review demonstrated low risk of bias.

**Table 2:** Quality of Included Randomized Controlled Trials (low, high, unclear)

Source	Selection bias	Performance bias (blinding of participants and staff)	Detection bias (blinding of outcome assessment)	Attrition bias (incomplete outcome data)	Reporting bias (selective reporting)	Other bias (important concerns)
Aspy et al., 2008 [25]	Low risk	Low risk	Low risk	Low risk	Low risk	None
Bertoni et al. 2009 [26]	Low risk	Low risk	Low risk	Low risk	Low risk	None
Clyne et al., 2015 [30]	Low risk	Low risk	Low risk	Low risk	Low risk	None
Dickinson et al., 2014 [18]	Low risk	Low risk	Low risk	Low risk	Low risk	Differences in baseline data
Dignan et al., 2014 [28]	High risk	Low risk	Low risk	High risk	High risk	FS and DCBE rates not reported
Engels et al., 2006 [19]	Low risk	High risk	High risk	Low risk	Unclear	Unblinded outcome assessment
Feldstein et al., 2006 [29]	Low risk	Low risk	Low risk	Low risk	Low risk	Differences in baseline data
Hennesy et al., 2006 [31]	Low risk	Low risk	Unclear	Low risk	Low risk	None
Hogg et al., 2008 [36]	Low risk	Low risk	Low risk	Low risk	Low risk	None
Lowrie et al., 2014 [22]	Low risk	Low risk	Low risk	High risk	Low risk	Differences in baseline data
Magrini et al., 2014 [23]	Low risk	Low risk	Low risk	High risk	Low risk	None
Mold et al., 2008 [17]	Low risk	Low risk	Low risk	Low risk	Low risk	Differences inbaseline data
Mold et al., 2014 [32]	Low risk	Low risk	Low risk	Low risk	Low risk	None
Naughton et al., [21]	Low risk	Unclear	Unclear	Low risk	Low risk	None
Ornstein et al., 2010 [33]	Low risk	Low risk	Low risk	Low risk	Low risk	None
Ornstein et al., 2013 [34]	Low risk	Low risk	Low risk	Low risk	Low risk	Practice selection
Parchman et al., 2013 [20]	Low risk	Low risk	Low risk	Low risk	Unclear	Practice selection
Rognstad et al., 2013 [24]	Low risk	Low risk	Low risk	Low risk	Unclear	Control group bias
Sheffer et al., 2012 [27]	Low risk	Low risk	Low risk	Low risk	Low risk	No cessation data
Smidth et al., 2013 [35]	Low risk	Low risk	Low risk	Low risk	Low risk	None
Varonen et al., 2007 [13]	Low risk	Unclear	Unclear	Low risk	Low risk	Delays in

Risk of bias is assessed using Cochrane Collaboration’s tool for assessing risk of bias [38]. This tool provides criteria for rating the risk of bias within each domain as low, high, or unclear

### Measured intervention strategies

All studies except one that focused solely on the effects of academic detailing [21] investigated the impact of multifaceted interventions on practice change in healthcare practices. Nine studies investigated the effects of two-component interventions [10–13, 16, 18, 19, 21–26]. Others investigated three or more components. Sixteen studies out of the 21 included in this review used academic detailing (AD) as part of a multi-component intervention strategy [10, 13, 17, 21, 23–34]. Thirteen studies had a form of audit and feedback integrated into their intervention [17–19, 22–25, 28, 29, 32–35]. Eleven studies employed a type of practice facilitation or coaching during their interventions [17–20, 25, 27, 32–36]. All studies that included practice facilitation reported significant effects in one or more study outcomes. None of the studies that demonstrated ‘no effect’ in primary outcomes employed practice facilitation as a component of their intervention except two [9, 36] which showed significant change only in secondary outcomes.

Five studies reported having a form of information technology or system support (IT) [17, 26, 29, 30, 35]. Bertoni et al. [26], Clyne et al. [30] Feldstein et al. [29], and Mold et al. [17] utilized automated reminders that alerted clinicians when there was an error in the system. Smidth et al. [35] provided online forms or informational websites to participants. Only 2 studies utilized regular phone calls [27, 37] with sites. Both studies reported this strategy as not significantly effective. Two studies mailed educational materials to patients [30, 31]. Neither demonstrated a significant effect.

### Background of academic detailers and external change agents

Most of the studies employed pharmacists and pharmacologists to deliver the academic detailing [9–12, 21, 24, 30]. Physicians and nurses were also engaged as academic detailers [8, 13, 24, 26, 27, 29, 33, 34, 36]. In some studies, the investigators employed quality improvement experts who were trained in organizational change implementation [10, 17, 18, 20, 25, 32, 37]. These studies usually focused on change implementation and targeted practice facilitation as part of their intervention rather than delivering only academic detailing. All of the studies that included practice facilitation through external change agents demonstrated positive effect regardless of the change agent’s specific background [17–20, 25, 27, 32–36].

## Discussion

### Effectiveness of multifaceted interventions

Because the context of each study varied and there was not a uniform reporting measure of effect size, we measured the effectiveness of an intervention based on the *p* values of change between study arms reported in each study. Thirteen of the 21 studies reported a significant positive change (*p* < .05) in their primary outcomes on the intervention arm [17–20, 22, 23, 25, 27–29, 32, 34, 35]. All of these studies investigated the effects of multi-faceted interventions that included at least two of the intervention categories described under the data analysis section.

Four studies reported that the intervention demonstrated mixed results; there was significant increase in several outcomes whereas there was no improvement in other outcomes. Bertoni et al. [26] reported that two of their four outcome measures had significant improvements. This study tested the effectiveness of academic detailing coupled with system support. Clyne et al. [30] reported a significant decrease in one of the primary outcomes whereas the improvement in second outcome was not significant. Mold et al. [17] reported significant positive impact in one of the three primary outcomes. Ornstein et al. [33] reported positive change in only a specific age group.

### Importance of individualized follow up

Follow-up individualized to the clinic stands out as an integral component of multi-faceted interventions for organizational change. A majority of the studies that reported significant improvement in their results included individualized follow up in their interventions. Studies use a number of titles to describe a change agent. These titles include practice facilitator, practice enhancement assistant, and outreach visitor. Facilitation is always conducted by an expert change agent who is trained in quality improvement tools and methods. The change agents perform the follow-up meetings in-person, and on a regular basis that averages a minimum of once a month. The changes that are implemented in the clinics are always tailored to clinic needs. Only one study that included change agents demonstrated no change [36].

Overall, six of the studies reported that their intervention did not demonstrate any significant improvement on the primary outcomes [13, 21, 24, 28, 31, 36]. One thing that these studies had in common was that all of them except one [36] tested academic detailing coupled with audit and feedback with little to no follow up, which strengthens the importance of facilitation on organizational change. A number of reasons were listed as possible cause for no effect. Hennesy et al. [31] emphasized that lack of intensive follow up may have caused the unfavorable outcomes. Varonen et al. [13] pointed out that some of the practices were already implementing the targeted policies of the intervention at baseline, limiting their outcomes. Two studies [21, 24] that showed no significant difference between study arms reported that both intervention and control groups demonstrated positive impact, which may be attributed to effects of larger scale campaigns taking place simultaneously with the study.

This review should be considered in the context of some limitations. Although our search results include a number of studies that reported no significant effect, it is possible that other clinical trials that did not demonstrate significant effects have not been published, and hence not included in this review; publication bias may have decreased the number of studies with negative findings. A second limitation is that variation in the types of labels used to identify the components of the interventions can act as a barrier to identifying relevant studies. For instance, practice facilitation can be labeled as coaching, consulting, or mentoring, whereas academic detailing can also be referred to using a wide set of terms, including learning collaboratives or problem-based learning. A third limitation is that our systematic review was rather tightly circumscribed through the application of our search criteria (we limited our search to randomized controlled trials featuring the use of external change agents in primary care settings in developed countries). We sought to minimize heterogeneity in terms of settings, study designs, and interventions in order to clearly isolate the effect of external change agents. This decision does not imply that there is not significant knowledge to be gained by studying the role of external change agents in developing countries, in healthcare settings besides primary care, and in otherwise eligible primary care studies that have used non-RCT study designs. Examination of the role of external agents across different contexts should be the subject of future research. Finally, the type, duration, and intensity of follow up (in-person or over the phone) provided through the practice facilitation is not always clearly reported, which may have an effect on the reported outcomes. The studies also vary in terms of how the educational materials are delivered (face to face vs online or mailed). This information was not always explicit in the reports.

## Conclusions

This systematic review outlines the characteristics and effectiveness of implementation strategies led by external change agents to promote improvement in healthcare organizations. Our review suggests important findings and points out critical gaps in knowledge that require further investigation. As an implementation strategy, simply informing clinics of opportunities to improve (via audit and feedback) or advising them on what they should be doing (via educational materials or system-level supports) appears generally insufficient to change clinical practice. In-person education (or persuasion) delivered via academic detailing also is insufficient to change clinical practice in the absence of frequent, individualized follow up with clinics. Overall, our results suggest that a multi-faceted implementation strategy featuring regular, tailored follow up via practice facilitation is most likely to promote successful organizational change. Provision of educational materials, audit and feedback, and system support are often integral components of such a strategy, but those components cannot function as implementation strategies on their own. Our findings, consistent with theories on organizational change, suggest that a more comprehensive strategy is required to change clinical practice, involving the thoughtful selection and deployment of external change agents who work intimately with clinic sites as part of a multifaceted implementation strategy.
